# Anticonvulsant Activity of Pterostilbene in Zebrafish and Mouse Acute Seizure Tests

**DOI:** 10.1007/s11064-019-02735-2

**Published:** 2019-01-28

**Authors:** Dorota Nieoczym, Katarzyna Socała, Kinga Gawel, Camila V. Esguerra, Elżbieta Wyska, Piotr Wlaź

**Affiliations:** 1 Department of Animal Physiology, Institute of Biology and Biochemistry, Faculty of Biology and Biotechnology, Maria Curie-Skłodowska University, Akademicka 19, 20-033 Lublin, Poland; 2 Chemical Neuroscience Group, Centre for Molecular Medicine Norway, University of Oslo, Gaustadalléen 21, 0349 Oslo, Norway; 3 Department of Experimental and Clinical Pharmacology, Medical University of Lublin, Jaczewskiego 8b, 20-090 Lublin, Poland; 4 Department of Pharmacokinetics and Physical Pharmacy, Collegium Medicum, Jagiellonian University, Kraków, Poland

**Keywords:** Pterostilbene, Seizures, Depression, Side effects, Zebrafish, Mice

## Abstract

Pterostilbene (PTE), a natural dimethylated analog of resveratrol, possesses numerous health-beneficial properties. The ability of PTE to cross the blood–brain barrier raised the possibility that this compound may modulate central nervous system functions, including seizure activity. The aim of our study was to investigate the activity of PTE in the larval zebrafish pentylenetetrazole (PTZ) seizure assay and three acute seizure tests in mice, i.e., in the maximal electroshock seizure threshold (MEST), 6 Hz-induced psychomotor seizure threshold and intravenous (*iv*) PTZ tests. Additionally, potential antidepressant activity of PTE was estimated in the forced swim test in mice. The chimney test was used to determine the influence of PTE on motor coordination in mice, while its influence on neuromuscular strength was assessed in the grip strength test in mice. Locomotor activity was determined to verify the results from the forced swim test. PTE revealed an evident anticonvulsant effect both in zebrafish larvae (10 µM; 2 h-incubation) and mice (at doses of 100 and 200 mg/kg, intraperitoneally) but it did not exhibit antidepressant potential in the forced swim test. Furthermore, it did not cause any statistically significant changes in motor coordination, neuromuscular strength and locomotor activity in mice. In conclusion, our present findings demonstrate for the first time the anticonvulsant potential of PTE. The aforementioned results suggest that it might be employed in epilepsy treatment, however, further precise studies are required to verify its activity in other experimental seizure and epilepsy models and its precise mechanism of action should be determined.

## Introduction

Epilepsy is a common chronic disorder which affects about 65 million people worldwide [1]. It is characterized by recurrent seizures which result from excessive and synchronous electrical activity of some groups of neurons in the central nervous system [1]. Since the introduction of the first antiepileptic drug—bromide, in 1857, nearly 40 antiepileptic agents have been entered into the pharmaceutical market. Despite high availability of antiepileptic medication approximately 30% of patients with epilepsy do not achieve satisfying effects of pharmacological treatment due to continued seizures and/or unacceptable side effects [2]. Furthermore, epidemiological studies revealed that 9–37% patients with epilepsy suffer also from depression and prevalence of depression is even higher in people with drug-resistant epilepsy [3]. Thus, there is a need to search new drugs with better efficacy and safety profile to treat patients with epileptic disorders alone and concomitant depression. Plants have been used for centuries and are still used in folk medicine and seem to be a rich source of new compounds with therapeutic properties, including anticonvulsant and/or antidepressant activity.

Pterostilbene (PTE; *trans-*3,5-dimethoxy-4′-hydroxy-trans-stilbene), a natural dimethylated analog of resveratrol (RES), was initially isolated from sandalwood and is also found in blueberries, grapes and peanuts [4]. Both of these compounds belong to stilbenoids which are synthesized in plants in response to stress conditions and/or some infections [5, 6]. Therapeutic effects of RES, the most well-known stilbene, have been intensively studied for over 20 years. PTE properties are much less known although it has recently received much attention from the scientific community because of its diverse health-beneficial properties, i.e., cardioprotective, antidiabetic, antioxidant, anti-inflammatory, analgesic and anticarcinogenic [5, 7]. Due to greater lipophilicity, PTE exhibits higher bioavailability and more easily crosses through the blood–brain barrier than RES and therefore it might be more efficacious in affecting activity of the central nervous system. It was revealed that PTE improved cognition and neuronal function in aging/Alzheimer murine models [6] as well as showed anxiolytic activity in the elevated plus maze test in mice [8].

The aim of the present study was to investigate anticonvulsant and antidepressant properties of PTE in animal models. Anticonvulsant activity was firstly estimated in the larvae zebrafish pentylenetetrazole (PTZ) seizure assay and afterwards it was investigated in three seizure models in mice, i.e., in the maximal electroshock seizure threshold (MEST), 6 Hz-induced psychomotor seizure threshold and intravenous (*iv*) PTZ tests. Furthermore, a median anticonvulsant doses of PTE (ED_50_, i.e., dose which inhibits occurrence of seizures in 50% of the tested animals) were determined in the 6 Hz-induced psychomotor seizure test. The used murine models were helpful in identifying majority of the currently marketed drugs for treatment of epilepsy and, although they were introduced over 60 years ago and possess a lot of disadvantages, they are still widely used in the preclinical screening of substances with the potential therapeutic properties. The zebrafish models have arisen during the last decade as an alternative to the previously existing rodent models of neurological diseases and they are also a valuable tool for looking for mechanisms of seizure discharges and new antiepileptic drugs. Most recently, the zebrafish larvae PTZ seizure assay is used for preliminary screening of compounds with potential anticonvulsant properties [9–11]. Antidepressant properties of PTE were studied using the forced swim (Porsolt) test—one of the most common tests used to assess depressive-like behavior in mice [12]. Additionally, to determine some possible acute side effects of PTE we evaluated its influence on the muscular strength and motor coordination in mice using the grip strength and chimney tests, respectively. We investigated also influence of PTE on the locomotor activity of mice to verify results from the forced swim test.

## Materials and Methods

### Zebrafish

Adult zebrafish (*Danio rerio*) stocks of AB strain were maintained under standard aquaculture conditions, i.e., 28.5 °C, on a 14/10 h light/dark cycle. Fertilized eggs were collected via natural spawning. Embryos were reared under constant light conditions in embryo medium, i.e., Danieu’s buffer: 1.5 mM Hepes, pH 7.6, 17.4 mM NaCl, 0.21 mM KCI, 0.12 mM MgSO_4_ and 0.18 mM Ca(NO_3_)_2_. All embryos and larvae were kept in an incubator at 28.5 °C. For all experiments, larvae of 117 h post-fertilization (hpf) were used.

### Mice

Male Swiss mice weighing 23–28 g (6–8 weeks old) obtained from a licensed breeder (Laboratory Animals Breeding, Słaboszów, Poland) were used in the study. We used male mice to avoid sex differences and influence of female estrus cycle. The animals were housed in the polycarbonate cages under strictly controlled conditions (ambient temperature 20–23 °C, relative humidity 45–65%, a 12/12 light/dark cycle with the light on at 6:00 a.m., chow pellets and tap water continuously available). Mice were used in the study after at least 1 week of acclimatization. All experiments were performed at the same time of day (between 8:00 a.m. and 3:00 p.m.) to minimize circadian influences. Control and drug experiments were always done on the same day to avoid day-to-day variations in convulsive susceptibility. Each mouse was used only once. All procedures were conducted in accordance with the European Union Directive of 22 September 2010 (2010/63/EU) and Polish legislation acts concerning animal experimentations. The experimental procedures and protocols were approved by the Local Ethics Committee in Lublin (18/2018 and 93/2018).

### Drugs

The following drugs were used: PTE (Toronto Research Chemicals INC, Toronto, ON, Canada), valproic acid (VPA, as sodium salt; Sigma–Aldrich Co., St. Louis, MO, USA) and imipramine (IMI; kindly donated by Polfa, Kraków, Poland). In the zebrafish larvae experiments, PTE was dissolved in DMSO to achieve final concentration 0.054% w/v. Embryo medium with 0.054% w/v concentration of DMSO was used as a vehicle control. PTZ was dissolved to 60 mM (3x stock) in embryo medium. For murine studies, all the used solutions/suspensions were administered intraperitoneally (*ip*) at a constant volume of 10 ml per kg body weight. VPA was dissolved in saline while PTE and IMI were suspended in 5% solution of Tween 80 (POCH, Gliwice, Poland) in normal saline. VPA was injected 15 min before the respective experimental procedure, while PTE and IMI 30 min before the tests. The pretreatment time for PTE was determined based on the results from pharmacokinetic studies and the pilot experiment that evaluated time-course effect of this compound in the *iv* PTZ test in mice, while pretreatment times for VPA and IMI were taken from the literature and confirmed in our previous studies. Mice in the negative control groups were treated with the respective vehicles at the appropriate volume and time.

### Toxicological Assessment in the Zebrafish Larvae

Maximum tolerated concentration (MTC) was evaluated prior to further experiments. Briefly, 4 dpf zebrafish larvae (n = 12/group) were incubated with a range of PTE doses at 28.5 °C, for 18 h. The following parameters were scored after 2 and 18 h of exposure: touch response, posture, edema, morphology, signs of necrosis, swim bladder and heartbeat. The highest concentration of PTE (i.e., 10 µM) that did not cause death of larvae or any signs of toxicity was chosen as MTC.

### Behavioral Analysis of Anticonvulsant Activity in Zebrafish

The locomotor activity of larvae was assessed as previously described [13]. A single 117 hpf zebrafish larvae was placed in a 48-well plate (one larva per well) filled with 200 µl of vehicle or PTE solution (10 µM). Subsequently, larvae were incubated for 2 h, at 28.5 °C. Next, 100 µl of vehicle or PTZ was added to each well. Within 5 min, plates were positioned in an automated tracking device (ZebraBox, Viewpoint, Lyon, France) and larvae were tracked for 30 min, with a 5 min integration interval. All measurements were performed at the same daytime period. The large distance (in millimeters) covered by each larva was recorded. Three independent experiments were done (n = 12/group) and the data were pooled together. Two-way with/without repeated measures analysis of variance (ANOVA) followed by the Bonferroni’s post-hoc test was used to analyze the data and p values equal to or lower than 0.05 were considered as a statistically significant difference. The data were normalized against PTZ values.

### Electrographic Discharge Assessment in Zebrafish

For local field potential (LFP) recordings, zebrafish larvae were incubated for 2 h, as described above. After incubation, larvae were exposed to vehicle or 20 mM PTZ for 5 min. Thereafter, larvae were immobilized in a thin layer of 2% low-melting-point agarose and the glass electrode (resistance 1–5 Ω) filled with artificial cerebrospinal fluid (124 mM NaCl, 2 mM KCl, 2 mM MgSO_4_, 2 Mm CaCl_2_, 1.25 mM KH_2_PO_4_, 26 mM NaHCO_3_, 10 mM glucose) was placed into the optic tectum (MultiClamp 700B amplifier, Digidata 1440A digitizer, Axon instruments, USA) [13, 14]. Single recordings for each larva were performed for a period of 20 min. The discharges were analyzed according to the duration of spiking paroxysms and only those where the amplitude exceeded three times the background noise were taken into account. The data were analyzed with the aid of Clampfit 10.2 software (Molecular Devices Corporation, USA).

### Determination of PTE in Mouse Serum and Brain Tissue and Pharmacokinetic Analysis

In order to isolate PTE from biological samples, brains were homogenized in distilled water (1:4, w/v) with a tissue homogenizer TH220 (Omni International, Inc., Warrenton, VA, USA). Aliquots of 0.2–0.3 ml of serum or 0.5–1 ml of brain homogenate were spiked with 10 µl of internal standard (IS) solution containing clonazepam at concentration of 10 µg/ml and vortexed (Reax top, Heidolph, Germany) for 10 s. Both serum samples and brain homogenates were extracted with 3 ml of a mixture of ethyl acetate:hexane (30:70, v/v) for 20 min on a shaker (VXR Vibrax, IKA, Germany). After centrifugation (Universal 32, Hettich, Germany) at 3000 rpm for 15 min, the organic layers ware transferred to new tubes and evaporated to dryness at 37 °C under a gentle stream of nitrogen. The residues were dissolved in 100 µl of methanol and aliquots of 10–50 µl of the solution were injected into the HPLC system. The analysis was performed on a 250 × 4.6 mm LiChrospher®100 RP-18 column with a particle size of 5 µm (Merck, Darmstadt, Germany) protected with a guard column (4 × 4 mm) with the same packing material. The mobile phase was composed of deionized water and acetonitrile mixed at a ratio of 50:50 (v/v) and pumped at a flow rate of 1 ml/min. The column temperature was maintained at 21 °C. The HPLC system (Merck-Hitachi, Darmstadt, Germany) consisted of an L-7100 isocratic pump, an L-7200 autosampler, and a UV variable-wavelength K-2600 detector (Knauer, Berlin, Germany) operating at 321 nm. D-7000 HSM software was used for data acquisition and processing. In these conditions, the retention times of IS and PTE were approximately 5.3 and 12.8 min. The calibration curve constructed by plotting the ratio of the peak area of PTE to IS vs PTE concentrations was linear in the tested concentration ranges. The limit of quantification of the analytical method was 5 ng/ml (or ng/g tissue). No interfering peaks were observed in the chromatograms. The method was precise and accurate with the intra- and inter-day coefficients of variation less than 10% and the accuracy expressed as percentage of the nominal concentration in the range of 95–109%. The extraction efficiencies of PTE and IS were higher than 90%.

Serum and total brain concentrations of PTE were determined in groups consisted of 8 animals. Pharmacokinetic parameters were calculated using non-compartmental analysis in Phoenix WinNonlin v. 7.0 (Pharsight Corporation, a *Certara* Company, Princeton, NJ, USA).

### The Timed *iv* PTZ Test in Mice

Each mice was placed in the cylindrical restrainer and the needle (27G, 3/4 in., Sterican®, B. Braun Melsungen AG, Melsungen, Germany) was injected into the lateral tail vein. The needle was attached by the polyethylene drain (PE20RW, Plastic One Inc. Roanoke, VA, USA) with the syringe containing 1% solution of PTZ (Sigma–Aldrich, St. Louis, MO, USA) in water and the syringe was located in the infuse pomp (model Physio 22, Hugo Sachs Elektronik–Harvard Apparatus GmbH, March-Hugstetten, Germany). The correct placement of the needle in the vein was confirmed based on the presence of the blood in the drain and the needle was protected against displacement by a peace of adhesive tape. Following injection, mice were took out from the restrainer and placed in the Plexiglas box for observation. The PTZ solution was infused into the vein at a constant rate of 0.2 ml/min. The time intervals from the start of infusion of PTZ solution to the beginning of three kinds of seizures, i.e., the first myoclonic twitch, generalized clonus with loss of the righting reflex and forelimb tonic extension, were noted. Infusion of PTZ solution was stopped immediately after onset of the tonic seizures, which usually were lethal. If animal survived the test, it was euthanized immediately. The threshold dose of PTZ (in mg/kg body weight) for each kind of seizures was calculated according to the following formula:

$${\text{PTZ~}}\left( {{\text{mg}}/{\text{kg}}} \right)=\frac{{{\text{infusion~duration~}}\left( {\text{s}} \right){\text{~}} \times {\text{~infusion~rate~}}\left( {{\text{ml}}/{\text{s}}} \right){\text{~}} \times {\text{~PTZ~concentration~}}\left( {{\text{mg}}/{\text{ml}}} \right)}}{{{\text{weight~}}\left( {{\text{kg}}} \right)}}$$

Experimental groups consisted of 9–13 animals. The data obtained in the *iv* PTZ test are presented as the threshold dose of PTZ (in mg/kg) + standard error of the mean (SEM) needed to elicit the respective kind of seizures. The data were analyzed by one-way ANOVA followed by the Tukey’s multiple comparison test. Statistical significance was noted when p values were equal or lower than 0.05.

### Maximal Electroshock Seizures Threshold Test

Seizures in the MEST test were evoked by applying a sine-wave alternating current (maximal output voltage 500 V, 50 Hz for 0.2 s) generated by constant current stimulator (Rodent Shocker, Type 221, Hugo Sachs Elektronik, Freiburg, Germany). The stimulation was delivered by saline-socked transcorneal electrodes. Before the stimulation 1% solution of tetracaine hydrochloride was applied into animals’ eyes for local anesthesia. During the stimulation animals were restrained manually and immediately after the stimulation they were placed in the Plexiglas box for observation. The endpoint evaluated in the test was the maximal electroconvulsion which was defined as extension of the hindlimbs above a 90° angle to the torso of animal.

The threshold for maximal electroshock was determined according to the up-and-down method described by Kimball et al. [15]. The mice in the experimental group were subjected to the stimuli with different current intensities (range of 8–30 mA) which changes in 0.06-log steps. If stimulated mouse respond with seizures, the next mouse in the group was stimulated with current of intensity 0.06-log step lower than the previous one. If the mouse did not exhibit seizures, the next one was stimulated with a current of intensity 0.06-log step higher than the previous one. Groups of 20 stimulated animals were used to calculate the threshold current strength (CS_50_ in mA; the current strength which induces tonic hindlimb extension in 50% of the tested animals) with SEM.

CS_50_ values were compared with one-way ANOVA followed by the Tukey’s multiple comparison test.

### The 6 Hz Psychomotor Seizure Test

Psychomotor (limbic) seizures were induced by transcorneal stimulation (6 Hz, 0.2 ms rectangular pulse, 3 s duration) generated by a Grass S48 stimulator coupled with a constant current unit CCU1 (both from Grass Technologies, West Warwick, RI, USA). Before stimulation a drop of ocular anesthetic, 1% tetracaine hydrochloride solution, was applied into the animals’ corneas. The electrodes were soaked in saline to ensure the good electrical contact. During the stimulation animals were restrained manually and immediately after the stimulation were placed in a Plexiglas box for observation of presence or absence of the psychomotor seizures. These seizures were characterized by immobilization or stun, often associated with rearing, forelimb clonus, twitching of vibrissae and elevated tail (Straub-tail), lasting at least 10 s from the stimulation. Lack of the above symptoms or their cessation within 10 s from the stimulation was considered as absence of seizure activity.

In the present study two 6 Hz-induced psychomotor seizure tests were performed: (1) the 6 Hz psychomotor seizure threshold test which employed stimulation at varied current intensities, and (2) the 6 Hz psychomotor seizure test with supramaximal stimulation at 32 mA and 44 mA.

In the 6 Hz psychomotor seizures threshold test, threshold current intensity (CS_50_ in mA) was estimated in groups of 20 animals according to the above mentioned up-and-down method [15]. Each mouse was stimulated only once at any given current intensity which was lowered if the previous animal exhibited seizures, or raised if the previous animal did not respond with seizures.

The 6 Hz psychomotor seizure test with the supramaximal stimulation at current intensity of 32 and 44 mA was used to determine ED_50_ dose (in mg/kg; the dose which protects 50% of the tested animals form the psychomotor seizures) of PTE. Groups of animals (3–5 groups, 8 animal/group) were treated with the increasing doses of PTE and stimulated with the fixed supramaximal current intensity, i.e., 32 mA or 44 mA. The percent of animals protected from convulsive activity was noted and the log-probit method [16] was used to determine ED_50_ doses of the studied compound.

One-way ANOVA followed by the Tukey’s post-hoc multiple comparison test was used to analyze CS_50_ values and p values equal or lower than 0.05 were considered as statistically significant difference.

### Forced Swim Test

The forced swim test was carried out according to the method described by Porsolt et al. [12] to assess antidepressant properties of PTE. Mice were placed individually in a glass cylinder (25 cm high; 10 cm diameter) containing tap water to a depth of 11 cm. Water was maintained at temperature of 23–25 °C. Mice were permitted to swim for 6 min and duration of their immobility was recorded during the last 4 min of the test by a trained experimenter who was blinded to the treatment. Immobility time was recorded using cumulative stopwatches. Mouse was considered to be immobile when it was floating in the water without struggling and making no attempts to escape, it was making only these movements necessary to keep its head above the water. After each trial, the water in the cylinder was replaced with fresh water. Experimental groups consisted of 10 animals.

Data obtained in the forced swim test were expressed as the mean (in s) + SEM immobility time in each experimental group. Results were analyzed by one-way ANOVA.

### The Grip-Strength Test

The acute effect of PTE on the skeletal muscles strength in mice was determined in the grip strength test [17]. The apparatus for this test (BioSeb, Chaville, France) consisted of a steel wire grid (8 × 8 cm) connected to an isometric force transducer. Each mouse was lifted by tail and allowed to grasp the grid with its forepaws and then it was gently pulled backward until its released the grid. The maximal force in newtons (N) exerted by mouse before leaving the grid was recorded. The mean of three consecutive measurements for each animal was calculated and normalized to body weight (mN/g).

Experimental groups consisted of 12 animals. Results obtained in the grip-strength test were compared using one-way ANOVA followed by the Tukey’s post-hoc multiple comparison test.

### The Chimney Test

The chimney test [18] was performed to evaluate influence of PTE on motor coordination in mice. During the test mice had to climb backwards up the plastic transparent cylinder (inner diameter 3 cm and length 30 cm) with gravelly inside. The inability of mice to escape from the cylinder within 60 s was considered as an motor impairment. Results obtained in the test were presented as percent of impaired mice in groups of 10–12 animals and were compared with the Fisher’s exact probability test.

### Locomotor Activity

Spontaneous horizontal locomotor activity of mice was measured using an automated infrared beam-based system (IR Actimeter with SedaCom32 computer software, Panlab/Harvard Apparatus, Barcelona, Spain). The system is composed of black square arena (25 × 25 cm) enclosed by transparent walls (height 35 cm) and surrounded by the frame containing 16 × 16 infrared beams. Occlusions of the photo beams were recorded by a computerized system and the interruption counts were used as a measure of horizontal locomotor activity of mice. Locomotor activity of each mouse was monitored during 5 or 6 min. The number of animals in each experimental group was 10. The data obtained in the test were expressed as mean activity counts/5 min ± SEM and were analyzed with one-way ANOVA.

## Results

### Effect of PTE in the Larvae Zebrafish PTZ Seizure Assay

**Fig. 1:**
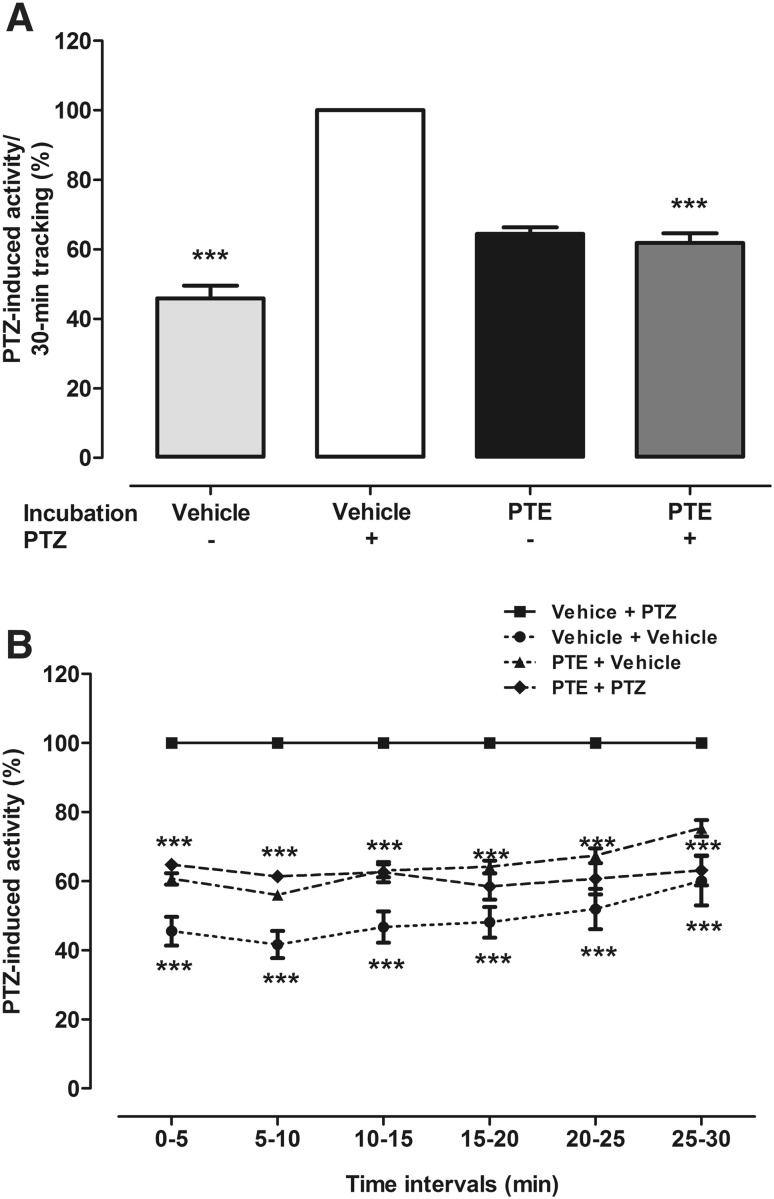
Effect of PTE on the seizure-like behavior in the zebrafish PTZ seizure assay. Zebrafish larvae after 2 h incubation with 10 µM PTE were exposed to 20 mM PTZ. Seizure-like behavior in the zebrafish larvae was measured 5 min after acute PTZ exposure. Results of the test are presented as total distance traveled during 30 min of session (**a**) and as total distance traveled per 5 min intervals (**b**). Data were analyzed using two-way ANOVA with/without repeated measures followed by the Bonferroni’s post-hoc test (n = 36/group). ***p < 0.001 vs. vehicle + PTZ group

**Fig. 2:**
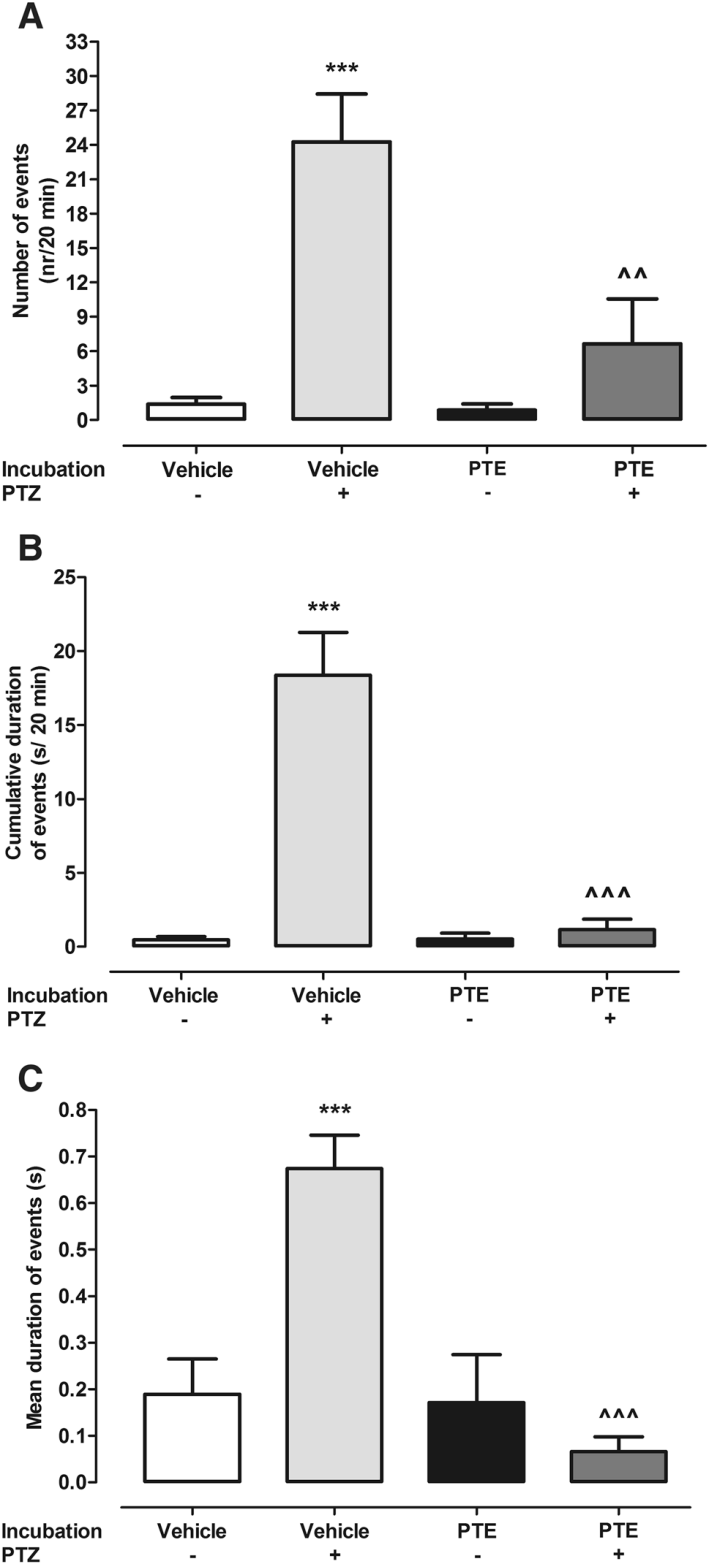
Electrophysiological recordings from the optic tectum of larvae pre-exposed to vehicle or PTE. Larvae were incubated with PTE (10 µM) for 2 h, and subsequently exposed to vehicle or PTZ (20 mM) for 5 min. The recordings started 5 min after the removal of each larva from PTZ solution and lasted 20 min. Results are presented as number of epileptiform events during 20 min (**a**), cumulative duration of epileptiform events during 20 min (**b**) and the mean duration of the event (**c**). Data were analyzed using two-way ANOVA followed by the Bonferroni’s post-hoc test (n = 8/group). ***p < 0.001 vs vehicle + vehicle group; ^^p < 0.01, ^^^p < 0.001 vs vehicle + PTZ group

PTE significantly reduced PTZ-induced locomotor activity in zebrafish larvae after 2 h incubation in its MTC, i.e., 10 µM (Fig. 1a, two-way ANOVA: F(1,137) = 130.62, p < 0.0001; Fig. 1b, two-way ANOVA with repeated measures: treatment × time interaction: F(15,700) = 4.47, p < 0.0001, treatment: F(3,700) = 63.18, p < 0.0001, time: F(5,700) = 13.16, p < 0.0001). The EEG recordings from the midbrain (optic tectum) of zebrafish larvae were performed to confirm anticonvulsant potential of PTE. Exposure to PTE significantly reduced both number (Fig. 2a, two-way ANOVA: F(1,28) = 9.76, p = 0.0041) and total duration (Fig. 2b, two-way ANOVA: F(1,28) = 32.36, p < 0.0001) of epileptiform discharges in the zebrafish brain in comparison to the control group which was incubated in the vehicle. Furthermore, there was also statistically significant reduction of the mean duration of ictal-like discharges (Fig. 2c, two-way ANOVA: F(1,28) = 33.32, p < 0.0001).

### Brain and Serum Concentration of PTE

Brain and serum concentration of PTE was determined after its acute *ip* administration to mice at a dose of 50 mg/kg.

As presented in Table 1, PTE was very fast absorbed from the peritoneal cavity and reached the peak concentration 15 min following compound dosing both in serum and brain. The compound very well penetrates into the brain of mice. The brain-to-serum C_max_ and AUC ratios were 6.0 and 5.15, respectively. The terminal half-life was relatively long in serum (2.64 h) and it was somehow shorter in the brain (1.82 h). This observation was further confirmed by the value of the mean residence time (MRT) in serum, which was over two times higher than in brain tissue.

**Table 1:** Brain and serum concentration of PTE in mice

Group	Concentration of PTE in brain tissue (µg/g)	Concentration of PTE in serum (µg/ml)
15 min	13.13 ± 1.82	1.76 ± 0.23
30 min	9.92 ± 1.10	1.08 ± 0.16
60 min	3.50 ± 0.44	0.55 ± 0.07
120 min	0.82 ± 0.14	0.21 ± 0.03
240 min	0.18 ± 0.06	0.10 ± 0.06

PTE was injected *ip* at a dose of 50 mg/kg. Each experimental group consist of 10 mice. Data are presented as mean ± SEM

**Fig. 3:**
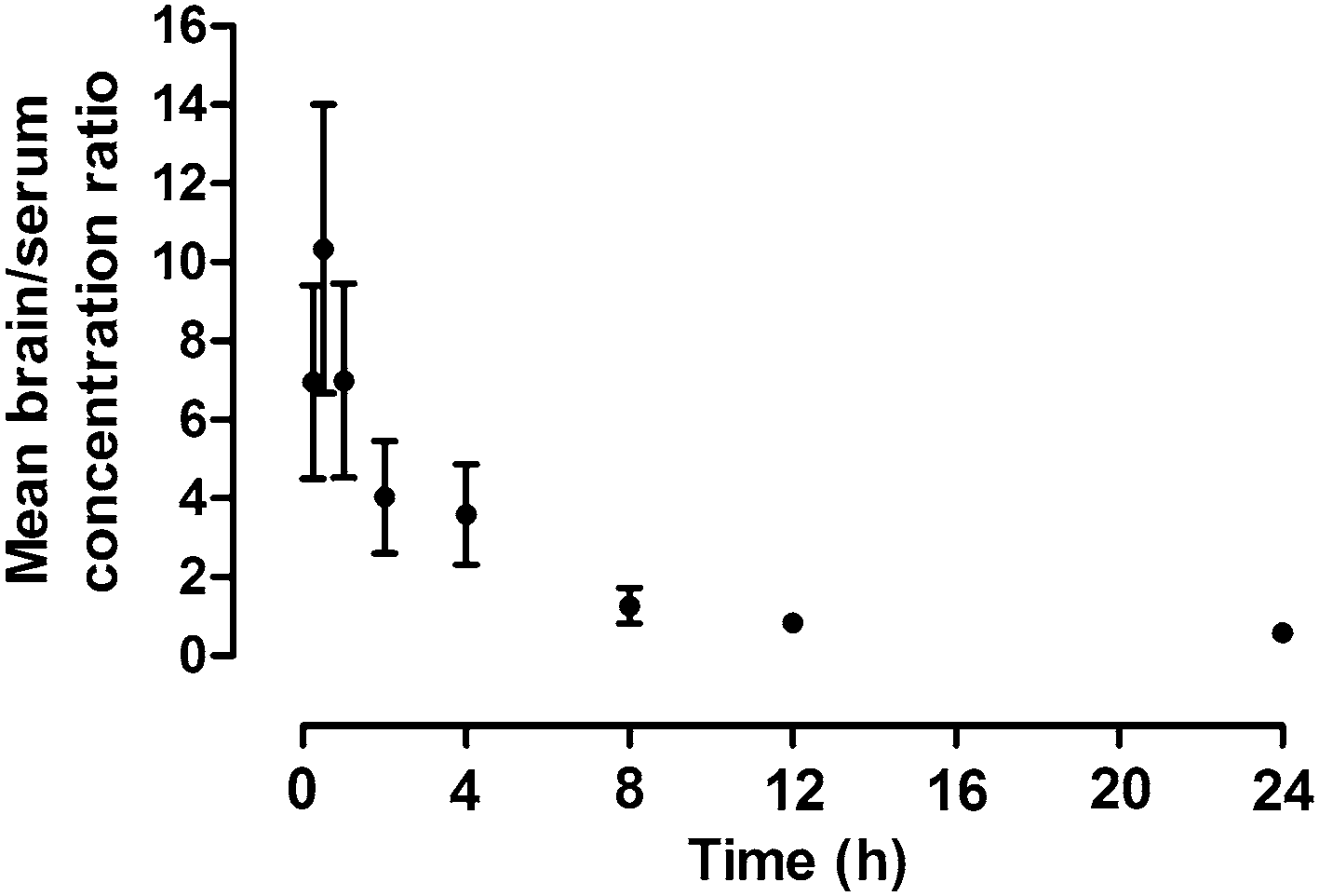
Mean brain-to-serum concentration ratio (± SEM) over time following *ip* administration of PTE at a dose of 50 mg/kg to mice (n = 8)

Pharmacokinetic parameters of PTE in mice following *ip* administration of a dose of 50 mg/kg are presented in Table 2. The mean brain/serum concentration ratios are presented in Fig. 3.

**Table 2:** Pharmacokinetic parameters of PTE in mice following *ip* administration of a dose of 50 mg/kg

Parameter	Estimate	
	Serum	Brain
t_max_ (h)	0.25	0.25
C_max_ (µg/ml or µg/g)	2.19	13.13
λ_z_ (h^−1^)	0.26	0.38
t_0.5 λz_ (h)	2.64	1.82
AUC_0–24_ (µg h/ml (g))	2.26	11.64
MRT (h)	2.62	1.09

C_max_ is the maximum concentration, t_max_ is the time to reach peak concentration, λ_z_ represents the terminal elimination rate constant, t_0.5λz_ is terminal half-life, calculated as ln2/λ_z_, AUC_0–24_ is area under the concentration–time curve from the time of dosing to the last measured point and MRT is the mean residence time calculated as AUMC_0–24_/AUC_0–24_, where AUMC is the area under the first moment curve

### Effect of PTE on Seizure Thresholds in the *iv* PTZ Test

**Fig. 4:**
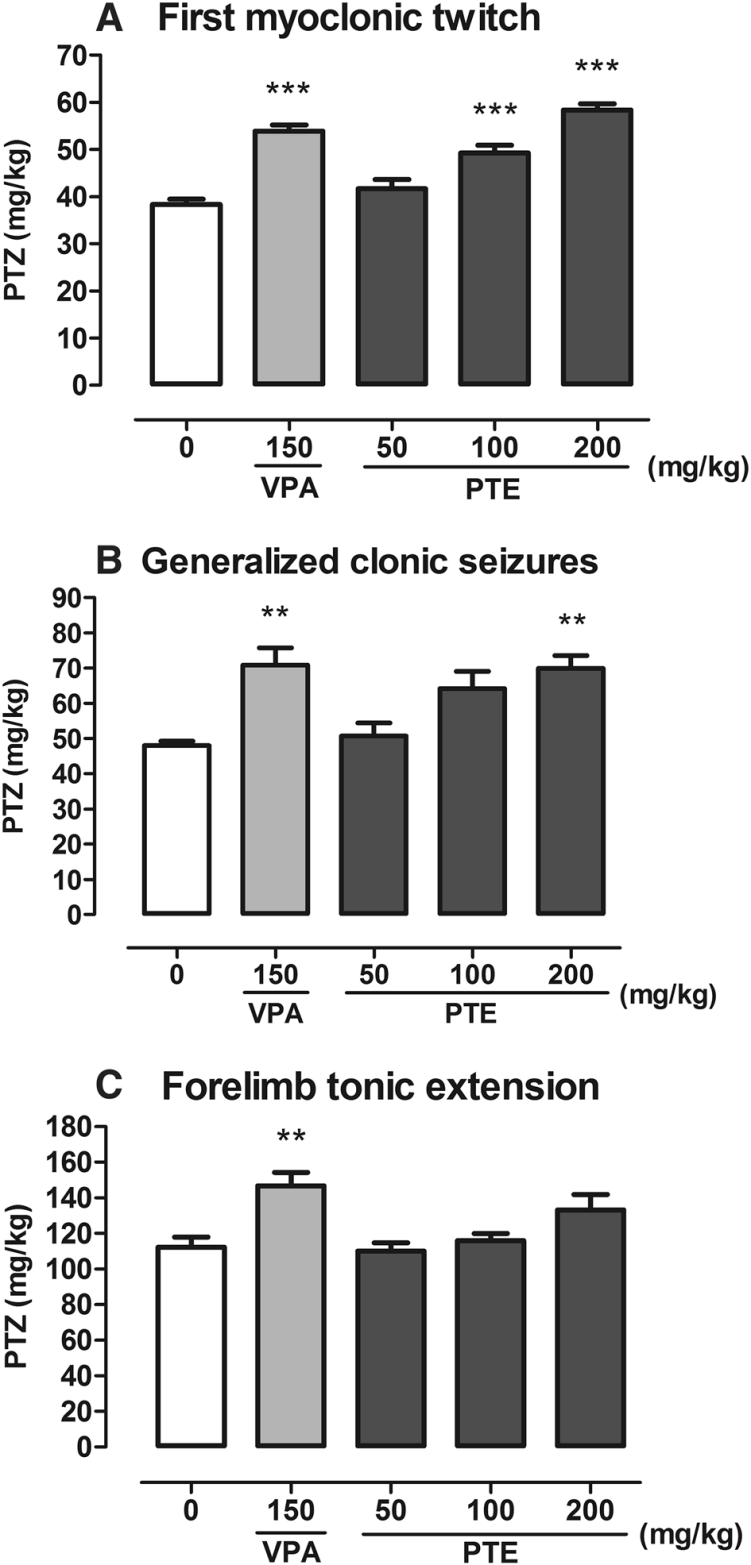
Effect of PTE on threshold for the first myoclonic twitch (**a**), generalized clonus (**b**) and forelimb tonus (**c**) in the *iv* PTZ test in mice. PTE (50–200 mg/kg) and VPA (150 mg/kg, positive control) were injected *ip* 30 and 15 min before the test, respectively. Negative control group received 5% Tween 80. Experimental groups consisted of 9–13 animals. Data are presented as mean (in mg/kg PTZ) + SEM and were analyzed using one-way ANOVA followed by the Tukey’s post hoc test, **p < 0.01, ***p < 0.001 vs the negative control group

PTE administered *ip* at doses range of 50–200 mg/kg significantly affected thresholds both for the first myoclonic twitch (one-way ANOVA: F(4,52) = 29.17; p < 0.0001) and generalized clonus with loss of righting reflex (one-way ANOVA: F(4,47) = 7.159; p = 0.0001) in the *iv* PTZ test in mice (Fig. 4a, b). Statistically significant increases in myoclonic seizure threshold were noted in groups of animals treated with PTE at doses of 100 and 200 mg/kg (p < 0.001) and in positive control (VPA-treated) group (p < 0.001). There were no statistically significant difference between groups treated with PTE (100 and 200 mg/kg) and VPA (150 mg/kg). In case of threshold for generalized clonic seizures statistically significant increase was noted both in group treated with VPA (p < 0.01) and PTE at a dose of 200 mg/kg (p < 0.01). In both of these groups, the thresholds were raised by over 45% in comparison to the negative control (5% Tween-treated) group. PTE at doses from 50 to 200 mg/kg did not significantly affect thresholds for tonic forelimb extension (Fig. 4c, one-way ANOVA: F(4,46) = 5.419; p = 0.0012) in the *iv* PTZ test in mice. Statistically significant increase in threshold for tonic seizures was noted only in group of animals treated with VPA (p < 0.01).

### Effect of PTE on Seizure Threshold in the MEST Test

**Fig. 5:**
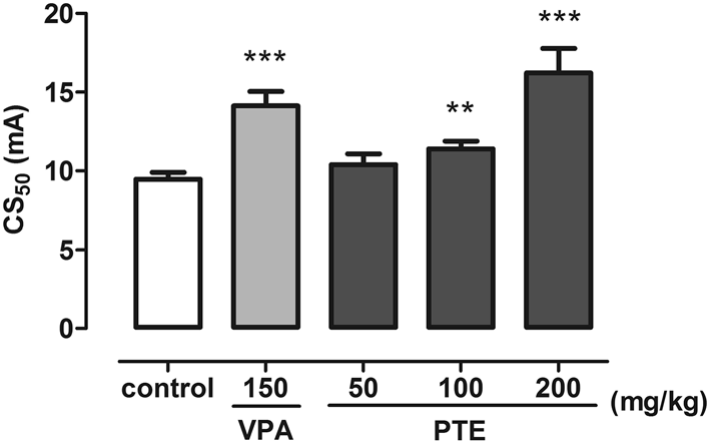
Effect of PTE on the seizure threshold in the MEST test in mice. PTE (50–200 mg/kg) and VPA (150 mg/kg, positive control) were injected *ip* 30 and 15 min before the test, respectively. Negative control group received 5% Tween 80. Experimental groups consisted of 20 animals. Data are presented as CS_50_ (in mA, current intensity which produce maximal electroshock in 50% of mice) with upper 95% confidence limits. Data were analyzed using one-way ANOVA followed by the Tukey’s post hoc test, **p < 0.01, ***p < 0.001 vs. the negative control group

The influence of PTE on seizure thresholds in the MEST test is presented in Fig. 5 (one-way ANOVA: F(4,43) = 49.28; p < 0.0001). CS_50_ value in the negative control (5% Tween-treated) group was 9.46 (9.04–9.90) mA while in group treated with VPA at a dose of 150 mg/kg it was raised to 14.13 (13.27–15.04) mA (~ 49%; p < 0.001). PTE injected at a dose of 100 mg/kg increased seizure threshold to 11.40 (10.93–11.87) mA (~ 20,5%; p < 0.01 in comparison to the negative control group). In group of animals treated with PTE at dose of 200 mg/kg seizure threshold was 16.22 (14.80–17.78) mA which was over 70% higher than in the negative control group (p < 0.001) and even 15% higher than in VPA-treated group (p < 0.05).

### Effect of PTE in the 6 Hz-Induced Psychomotor Seizure 6 Hz Test

**Fig. 6:**
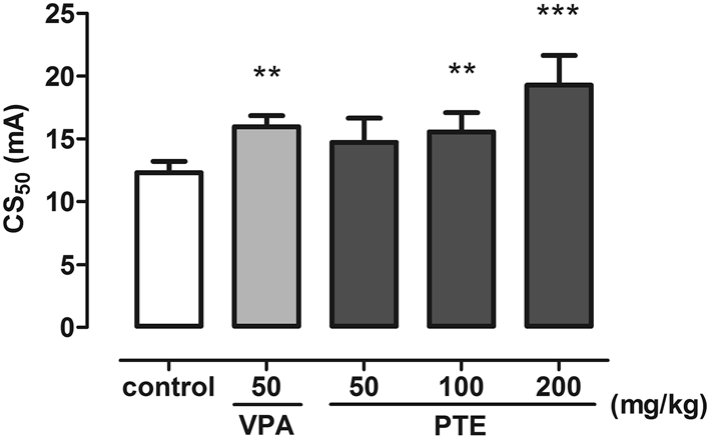
Effect of PTE on the seizure threshold in the 6 Hz test in mice. PTE (50–200 mg/kg) and VPA (50 mg/kg, positive control) were injected *ip* 30 and 15 min before the test, respectively. Negative control group received 5% Tween 80. Experimental groups consisted of 20 animals. Data are presented as CS_50_ (in mA, current intensity which produce psychomotor seizures in 50% of mice) with upper 95% confidence limits. Data were analyzed using one-way ANOVA followed by the Tukey’s post hoc test, **p < 0.01, ***p < 0.001 vs. the negative control group

Figure 6 presents the effect of PTE on the psychomotor seizure threshold in the 6 Hz test in mice (one-way ANOVA: F(4,41) = 10.53; p < 0.0001). The psychomotor seizure threshold in the negative control (5% Tween-treated) group was 12.30 (11.46–13.20) mA and it was raised to 15.97 (15.13–16.85) mA in group injected with VPA at a dose of 50 mg/kg. Statistically significant increases in the psychomotor seizure thresholds were noted also in groups treated with PTE at doses of 100 (p < 0.01) and 200 mg/kg (p < 0.001). CS_50_ values in these groups were 15.56 (14.18–17.08) mA and 19.28 (17.15–21.66) mA, respectively. There were no statistically significant differences in the seizure thresholds between VPA-treated and all PTE-treated groups (p > 0.05).

Because PTE considerably raised threshold for 6 Hz-induced psychomotor seizures we made an attempt to determine its ED_50_ dose in this test. When animals were stimulated with current intensity of 32 mA, ED_50_ value of PTE was 505.6 ± 44.7 mg/kg, and this value raised to 540.9 ± 41.5 mg/kg when current intensity was 44 mA.

### Effect of PTE in the Forced Swim Test

PTE at doses ranging from 50 to 200 mg/kg did not significantly reduce the immobility time in the forced swim test in mice (one-way ANOVA: F(4,45) = 5.755, p = 0.0008; data not shown). Statistically significant difference was noted only between control (5% Tween-treated) group and group of animals treated with IMI at a dose of 20 mg/kg (p < 0.05).

### Effect of PTE in the Chimney Test and Grip-Strength Test

Influence of PTE on the skeletal muscles strength and motor coordination is presented in Table 3. PTE administered at doses ranging from 50 to 800 mg/kg did not significantly influence muscular strength (one-way ANOVA: F(8,89) = 1.681, p = 0.1141) in mice. PTE (50–800 mg/kg) did not significantly influence motor coordination in the chimney test in mice (the Fisher’s exact probability test: p < 0.05).

**Table 3:** Influence of PTE on the neuromuscular strength and motor impairments in mice

Treatment (mg/kg)	Neuromuscular strength (mN/g)	Impairment of motor coordination (%)	N
Control (5% Tween)	30.17 ± 1.45	0	12
VPA (50)	31.12 ± 1.20	0	11
VPA (150)	25.36 ± 0.92	0	12
PTE (50)	30.34 ± 1.16	0	12
PTE (100)	29.14 ± 0.95	0	12
PTE (200)	28.76 ± 2.16	8	10
PTE (400)	30.39 ± 2.17	0	10
PTE (600)	30.19 ± 0.97	0	9
PTE (800)	31.12 ± 1.44	8	10
	*One-way ANOVA: F(8,89)* = *1.681, p* = *0.1141*		

VPA was administered 15 min and PTE 30 min before the tests. Control group received vehicle. All solutions/suspensions were injected *ip*. Results from the grip test are presented as the mean ± grip strengths in milinewtons per gram of mouse body weight (mN/g) and were analyzed with one-way ANOVA. Data from the chimney test are presented as a percentage of animals with impairment of motor coordination and were compared using the Fisher’s exact probability test

### Effect of PTE on the Spontaneous Locomotor Activity

Neither PTE administered at doses ranging from 50 to 200 mg/kg nor IMI at a dose of 20 mg/kg did not significantly change locomotor activity of mice (one-way ANOVA: F(4,44) = 2.294, p = 0.0743). Moreover, there were not statistically significant changes in locomotor activity between the control (5% Tween-treated) group and groups which were treated with PTE at high doses, i.e., 200–800 mg/kg (one-way ANOVA: F(4,44) = 7.0, p = 0.0002). Data are presented in Table 4.

**Table 4:** Influence of PTE (50–800 mg/kg) and IMI (20 mg/kg) on the spontaneous locomotor activity of mice

Treatment (mg/kg)	Activity counts	N
A. 6 min		
Control (5% Tween)	907 ± 131	10
IMI (20)	1217 ± 114	9
PTE (50)	857 ± 132	10
PTE (100)	747 ± 96	10
PTE (200)	874 ± 87	10
	*One-way ANOVA: F(4,44)* = *2.294, p* = *0.0743*	
B. 5 min		
Control (5% Tween)	1371 ± 141	10
PTE (200)	880 ± 134	10
PTE (400)	1603 ± 161	10
PTE (600)	806 ± 116	10
PTE (800)	1613 ± 181	9
	*One-way ANOVA* *F(4,44)* = *7.0, p* = *0.0002*	

PTE and IMI were injected *ip* 30 min before the test. Animals in the control groups received 5% Tween in saline. Results are presented as mean ± SEM. Data were analyzed using one-way ANOVA followed by the Tukey’s post hoc test

## Discussion

RES has attracted much scientific attention since 1980s, mainly due to a phenomenon known as the “French paradox”. Scientific research showed numerous beneficial health effects of this compound, i.e., anti-inflammatory, antioxidant, antimicrobial, cardioprotective, anticancer, antidiabetic and neuroprotective properties [19, 20]. However, the main disadvantages of RES are relatively poor solubility, low *in vivo* bioavailability and rapid metabolism after oral administration, which limits its clinical efficacy. PTE seems to be a compound with better pharmacokinetic properties and similar therapeutic effects. It also has better oral absorption and higher lipophilicity. Due to the more lipophilic nature and, thus, better blood–brain barrier permeability, PTE appears to be more efficacious in modulating brain functions than RES [20].

In our study, the anticonvulsant potential of PTE was first evaluated in the zebrafish PTZ seizure test [10, 13, 21] and this assay demonstrated its evident seizure-suppressing activity. Interestingly, exposure time of 2 h was sufficient to detect its bioactivity, whereas most of the compounds required longer, usually 18 h, incubation to exhibit its properties. This might be due to the properties of PTE since lipophilic compounds penetrate more easily into zebrafish larvae than the hydrophilic ones and their effects might therefore be unveiled already after short exposure [10, 11]. Furthermore, the ability of PTE to readily penetrate biological barriers was also confirmed by our results of pharmacokinetic experiments in mice because the highest concentration of the compound both in serum and brain was noted already 15 min after *ip* administration. A high level of PTE was maintained up to 30 min and rapidly declined at 1 h after the injection.

The ability of PTE to cross the blood–brain barrier was previously demonstrated in rodents [7, 8]. Azzolini et al. [7] noted that PTE peaked in brain tissue 2 h after oral administration and its concentration was nearly fivefold higher than pterostilbene-4’sulphate—the main metabolite of PTE, which was predominant in other tissues [7]. Therefore, it might be supposed that changes in central nervous system functions are caused by PTE itself, not by its metabolites. There are no studies concerning pharmacokinetic and tissue distribution of PTE after *ip* administration in mice and thus we could not compare our results precisely. Differences in time of peak concentration between our study and data presented by Azzolini et al. [7] might result mainly from the route of administration as substances injected *ip* enter the circulation faster than orally administered. Furthermore, we could not eliminate differences related to the injected doses and species of the experimental animals.

Anticonvulsant properties of PTE firstly estimated in the larval zebrafish PTZ assay were afterwards validated in the acute seizure tests in mice. At the beginning of our studies in mice, we determined the pretreatment time for PTE. Evaluation of time-course effect in the *iv* PTZ test revealed the highest anticonvulsant activity 30 min after its *ip* administration to mice (data not shown). This time did not correspond with the highest concentration of the studied stilbene in the brain. The noted delay of behavioral effect might be caused by activation and/or inhibition of some intracellular pathways that subsequently influence neurotransmission systems and decrease seizure vulnerability. Briefly, the pretreatment time for PTE in our study, i.e. 30 min, was determined based on the results of both pharmacokinetic and behavioral studies.

PTE significantly increased seizure thresholds in all the employed acute seizure tests in mice, i.e., the *iv* PTZ, MEST and 6 Hz psychomotor seizure threshold test. The MEST test is considered as model of generalized tonic–clonic seizures in humans and protective activity in this test show mainly drugs which inactivate the voltage sensitive sodium channels [22], while the PTZ test is thought to be useful to identify drugs effective in combatting generalized absence seizures in patients, in particular molecules which enhance GABA-ergic neurotransmission. The MEST test is an alternative method of the MES test and, in contrast to the MES test, it consults individual seizure sensitivity of animals and enables to detect anticonvulsant properties of drugs with GABA-ergic mechanism of action which usually are inactive or only weakly active in the conventional MES test with supramaximal stimulation [23]. The *iv* PTZ test is a very sensitive method which allow to study influence of the tested compound on the different components of the convulsant behavior and it is also useful in detection of the proconvulsant effects [22, 24, 25]. The third of the tests used – the 6 Hz test in mice, was initially described in the 1950s as model of psychomotor seizures but due to inactivity of phenytoin in this test it was recognized as unusable and abandoned for nearly 50 years. It was rediscovered after studies performed by Barton et al. [26] and afterwards was included in tests used in preclinical studies of compounds with potential anticonvulsant activity [22].

Anticonvulsant activity of PTE in the 6 Hz test was potent enough to determine its ED_50_ doses for seizures induced by supramaximal stimulation at 32 and 44 mA. Although PTE at a dose of 200 mg/kg raised seizure threshold in the MEST test above 70%, we did not manage to determine its ED_50_ dose in the MES test. In the *iv* PTZ test, PTE increased significantly seizure thresholds for the onset of the myoclonic twitches and generalized clonic seizures but it did not influence tonic seizure threshold. Greater ability to inhibit clonic seizures than tonic ones could be associated with different brain structures which generate these kinds of seizures since forebrain structures are characteristic for the clonic seizures, while tonic seizures originate from the brainstem structures [25]. Furthermore, the lack of significant anticonvulsant activity in the MES test suggests that PTE probably does not interact with sodium channels which are considered as the main targets for compounds which show high anticonvulsant activity in this test [23]. Additionally, significant anticonvulsant effect of PTE in the PTZ test might be evidence of interactions with GABA-ergic neurotransmission. Rueda et al. [27] demonstrated that PTE enhances GABA-induced chloride currents [27].

To our knowledge, the anticonvulsant properties of PTE have not yet been studied and only anticonvulsant properties of RES were presented previously in the rodent models. RES decreased incidence of the generalized tonic-clonic seizures [28] as well as reduced the duration of clonic seizures [29] in the PTZ test in rats. Interestingly, its anticonvulsant properties were not approved in the PTZ test in mice [30], which might be related to the differences in pharmacokinetics of the drug in these two animal species. RES showed significant anticonvulsant activity neither in the MES nor 6 Hz psychomotor seizure tests in mice [30].

Due to structural similarity, it is also likely that mechanism of action of PTE might be similar to RES and wherefore during further studies particular attention should be paid to some intracellular targets including, among others, AMP activated kinase (AMPK), Sirtuin (SIRT) system [31] and mammalian target of rapamycin (mTOR) pathway [32]. Since inflammatory processes and oxidative stress in the brain participate both in acute seizures and epileptogenesis [33–35], effect of PTE on these processes should also be investigated in the future studies. It was previously reported that PTE inhibits expression of pro-inflammatory cytokines, including THF-alpha, IL-1β, IL-6 [36]. Furthermore, Naik et al. [37] demonstrated that PTE improved antioxidant parameters in brain of rats treated with streptozotocin, i.e., increased catalase and superoxide dismutase activity as well as GSH level, reduced levels of nitrites, lipid peroxides and carbonylated proteins [37]. Further studies concerning anticonvulsant properties of PTE should also include its influence on the reactive gliosis and blood–brain barrier breakdown in seizure and epilepsy models.

In addition to the anticonvulsant effect, we also examined activity of PTE in the forced swim test in mice—one of the most frequently used model for screening drugs that may be used to treat depression. Immobility time, that is measured in this test, is an indicator of the behavioral despair and helplessness in experimental animals, higher immobility time indicates greater depressive behavior [38]. We did not reveal any statistically significant effect of PTE on the animal immobility in this test. Acute injection of PTE, both at doses employed in the forced swim test (i.e. 50–200 mg/kg) and at higher doses of 400–800 mg/kg, did not affect locomotor activity in mice which demonstrates that the lack of effect in the forced swim test was not due to the decrease in locomotor activity. Antidepressant potential of PTE has not been previously investigated but there are numerous studies which proved antidepressant activity of RES in both rodents and humans [39, 40]. Both acute and chronic administration of RES decreased immobility time in the forced swim test and did not affect locomotor activity in rats [41]. Moreover, RES reversed lipopolisaccharide- [42] and corticosterone-induced [43] depressive behavior in the forced swim test as well as revealed antidepressant action in other test used in the screening for new antidepressants [40, 44–46]. Although antidepressant properties of PTE were not show in the forced swim test, there is a great necessity to evaluate its activity in other experimental models of depressive-like behavior.

Taking antiepileptic drugs which are currently available on the pharmaceutical market is often associated with the occurrence of some adverse effects, i.e., impairment of motor coordination and/or memory and learning dysfunction [47]. To complement current knowledge about PTE and to assess safety of its biomedical use, we determined its influence on the motor coordination and muscular strength in mice. We did not detect any significant changes in the motor coordination that was assessed in the chimney test. There are no other studies that evaluated the acute effects of PTE on motor coordination in rodents but Li et al. [36] reported that PTE improved deficits in motor coordination in rats with neonatal hypoxic-ischemic brain damage. PTE also did not influence neuromuscular strength in the grip strength test in mice, which has not been evaluated previously. Our study confirmed the lack of severe toxic effects of PTE which was previously reported both in mice [48] and humans [49].

In conclusion, our findings demonstrated for the first time the anticonvulsant potential of PTE. High doses of PTE neither caused impairment of motor coordination and muscular strength nor influence locomotor activity in mice, which suggests that this compound may also be devoid of these side effects in humans. Natural compounds are widely used as a complementary alternative therapy in the treatment of numerous neurological diseases, also in epilepsy. Our results indicate that PTE might be employed in epilepsy treatment. However, further precise studies are required to verify its activity in other experimental seizure and epilepsy models and its precise mechanism of action should be investigated. Although we did not ascertain significant antidepressant activity of PTE in the forced swim test, it should be validated in other experimental tests for assessment of depressive-like behavior.
